# Impact of temperature on the extrinsic incubation period of Zika virus in *Aedes aegypti*

**DOI:** 10.1371/journal.pntd.0008047

**Published:** 2020-03-18

**Authors:** Olivia C. Winokur, Bradley J. Main, Jay Nicholson, Christopher M. Barker

**Affiliations:** 1 Department of Pathology, Microbiology and Immunology, School of Veterinary Medicine, University of California, Davis, California, United States of America; 2 Graduate Group of Entomology, University of California, Davis, California, United States of America; Institute for Disease Modeling, UNITED STATES

## Abstract

Since Zika virus (ZIKV) emerged as a global human health threat, numerous studies have pointed to *Aedes aegypti* as the primary vector due to its high competence and propensity to feed on humans. The majority of vector competence studies have been conducted between 26–28°C, but arboviral extrinsic incubation periods (EIPs), and therefore transmission efficiency, are known to be affected strongly by temperature. To better understand the relationship between ZIKV EIPs and temperature, we evaluated the effect of adult mosquito exposure temperature on ZIKV infection, dissemination, and transmission in *Ae*. *aegypti* at four temperatures: 18°C, 21°C, 26°C, and 30°C. Mosquitoes were exposed to viremic mice infected with a 2015 Puerto Rican ZIKV strain, and engorged mosquitoes were sorted into the four temperatures with 80% RH and constant access to 10% sucrose. ZIKV infection, dissemination, and transmission rates were assessed via RT-qPCR from individual mosquito bodies, legs and wings, and saliva, respectively, at three to five time points per temperature from three to 31 days, based on expectations from other flavivirus EIPs. The median time from ZIKV ingestion to transmission (median EIP, EIP_50_) at each temperature was estimated by fitting a generalized linear mixed model for each temperature. EIP_50_ ranged from 5.1 days at 30°C to 24.2 days at 21°C. At 26°C, EIP_50_ was 9.6 days. At 18°C, only 15% transmitted by day 31 so EIP_50_ could not be estimated. This is among the first studies to characterize the effects of temperature on ZIKV EIP in *Ae*. *aegypti*, and the first to do so based on feeding of mosquitoes on a live, viremic host. This information is critical for modeling ZIKV transmission dynamics to understand geographic and seasonal limits of ZIKV risk; it is especially relevant for determining risk in subtropical regions with established *Ae*. *aegypti* populations and relatively high rates of return travel from the tropics (e.g. California or Florida), as these regions typically experience cooler temperature ranges than tropical regions.

## Author summary

Understanding how the environment affects Zika virus transmission is critical for protecting public health. Warmer temperatures accelerate mosquitoes’ transmission of disease-causing pathogens, but this is not well-characterized for Zika virus in its primary vector, *Aedes aegypti*. In this study we measure the effect of a range of temperatures on Zika virus infection, dissemination, and transmission in *Ae*. *aegypti* and provide a mathematical equation that can be used in risk models to predict Zika virus transmission dynamics. We also explore how the effect of temperature on Zika virus transmission compares to the effects of temperature on other mosquito-borne viruses.

## Introduction

Zika virus (ZIKV) is a primarily mosquito-borne flavivirus that was first isolated in 1947 from a sentinel rhesus macaque in the Zika Forest of Uganda [1]. The virus has since spread beyond Africa, causing human outbreaks in the South Pacific in 2007 and 2013 [2,3]. In 2015, local transmission of ZIKV was first detected in Brazil [4]. ZIKV subsequently spread through much of the Americas, where transmission was detected in 48 countries in North and South America in 2016, including in the continental United States in Florida and Texas (CDC, PAHO). Symptomatic Zika disease typically involves fever, muscle and joint pain, conjunctivitis, and rash. At least 20% of infections are symptomatic, although estimates vary by study and subpopulation [2,5]. As ZIKV spread throughout the Americas, more severe manifestations were noted, including Guillain-Barre syndrome and microcephaly in neonates [6–8].

*Aedes aegypti* is the primary vector of ZIKV globally [9–11]. At present, the majority of ZIKV vector competence work in *Ae*. *aegypti* has been conducted at temperatures between 26–28°C [11–16]. Only one study has assessed vector competence outside of this range [17], even though *Ae*. *aegypti* has well-established populations that extend into subtropical regions such as California and Florida. Further, it is known that the arboviral extrinsic incubation period (EIP), the time from ingestion of virus until a mosquito is able to transmit, is strongly affected by temperature [17–20]. Most mechanistic models for Zika virus transmission use estimated EIP from other related viruses, most notably dengue, as the effect of temperature on ZIKV EIP has not been characterized until recently [17].

In this study, we conducted an experiment to quantify the relationship between ZIKV EIP and temperature, with the expectation that ZIKV transmission would accelerate at warmer temperatures. We were then interested in contrasting the EIP-temperature relationship for ZIKV with that of other flaviviruses of tropical and temperate origins. Our laboratory experiments were conducted with *Ae*. *aegypti* at four constant temperatures spanning the relevant range to which *Ae*. *aegypti* mosquitoes are likely to be exposed in nature. These results will be broadly applicable as *Ae*. *aegypti* is the most important vector of ZIKV globally. Results can be compared to the limited work on ZIKV EIP and can be incorporated into statistical models in order to understand geographical and seasonal ZIKV disease burden [17]. These results are of particular importance to subtropical regions such as Mexico, Florida, California, and Texas, where *Ae*. *aegypti* populations are already established and travelers frequently arrive from tropical locations that experienced Zika outbreaks.

## Materials and methods

### Ethics statement

This study was carried out in strict accordance with the UC Davis Institutional Animal Care and Use Committee (IACUC) Protocol #19404 that was reviewed and approved on June 29, 2017. The UC Davis IACUC adheres to the Office of Laboratory Animal Welfare Health Research Extension Act of 1985 (Public Law 99–158) as well as the United State Department of Agriculture’s Animal Welfare Act. UC Davis is accredited by the Association for Assessment and Accreditation of Laboratory Animal Care, International (AAALAC) and has an Animal Welfare Assurance (number A3433-01) on file with the Office of Laboratory Animal Welfare (OLAW).

### Mosquitoes, virus, and mice

*Ae*. *aegypti* mosquitoes colonized in 2016 from field-collected eggs in Clovis, California were used. The F4 generation used in this study was reared under standard, controlled conditions at 26°C, 80% RH, 12:12 L:D cycle with 200 larvae in one liter DI H_2_0 and one pinch fish food (c.a. 0.5 g, Tetramin) every other day until pupation. Pupated mosquitoes were transferred into a 30x30x30 cm mesh cage (BugDorm, MegaView Science Co., Taiwan) to emerge. Upon emergence, adult mosquitoes had constant access to 10% sucrose until 24 hrs before experimental use when sucrose was removed.

An Asian-lineage outbreak strain of ZIKV from Puerto Rico was used. The virus was first isolated from human serum during the outbreak in 2015 (PR15, PRVABC59), passaged four times in Vero cells, and sequenced. The coding sequence for the complete genome was identical to GenBank accession number KX601168. PR15 was obtained from Aaron Brault at the U.S. Centers for Disease Control and Prevention in Fort Collins, Colorado.

Female interferon-deficient (IFN-α/βR−/−; C57BL/6) mice aged five weeks (B6.129S2-Ifnar1tm1Agt/Mmjax, The Jackson Laboratory) were used.

### Mouse infections

**Fig 1:**
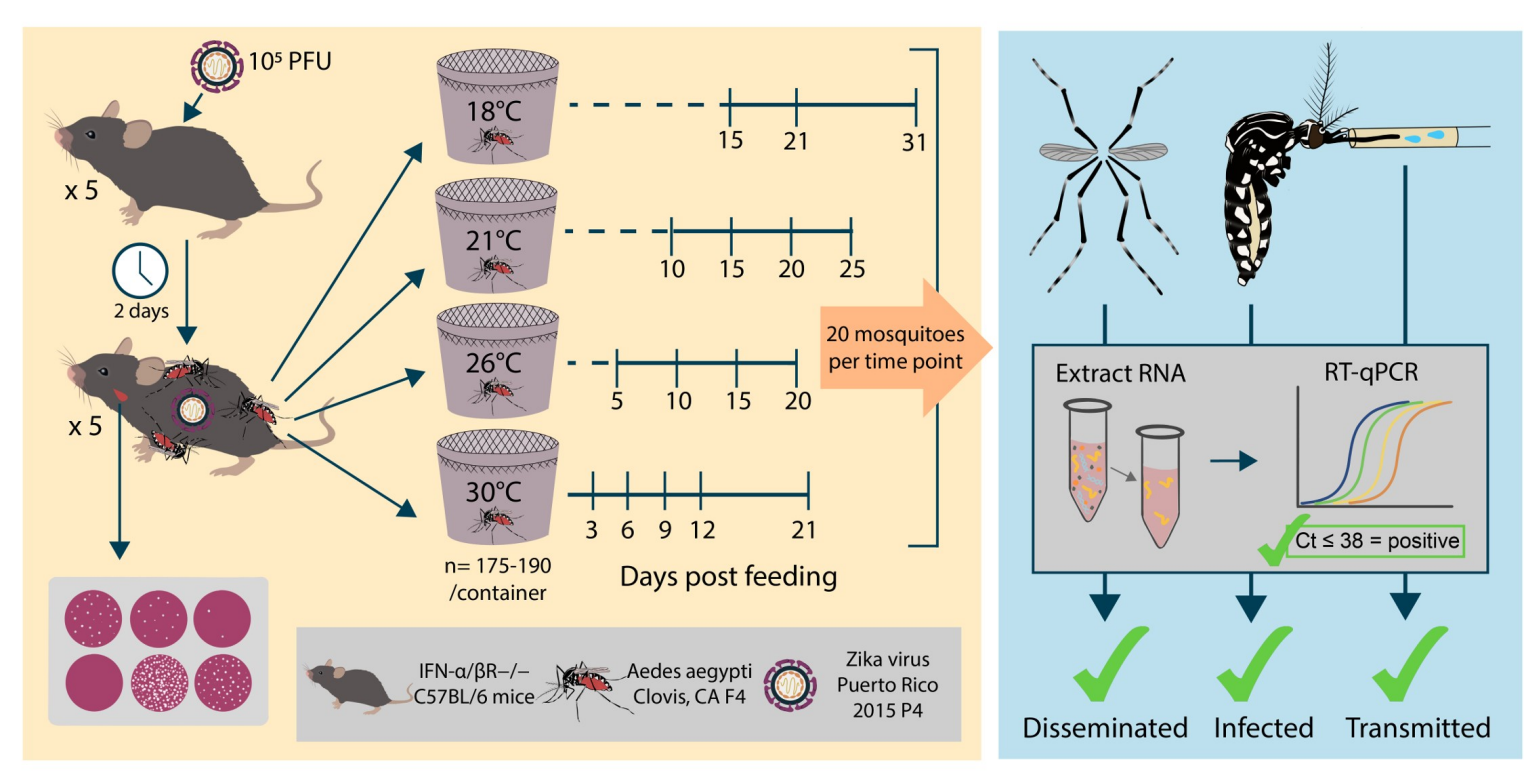
Study design workflow. Five five-week-old interferon knockout mice were inoculated with 10^5^ Vero PFU of ZIKV PR15 via subcutaneous injection and held for two days to reach peak viremia. After two days, mice were anesthetized, blood was collected via each mouse’s submandibular vein (i.e., cheek punch) and blood serum ZIKV titer was assessed using Vero cell plaque assay, and 1,200 female *Aedes aegypti* mosquitoes aged 3–5 days were exposed to all five mice. Bloodfed mosquitoes were randomly sorted into four half-gallon cartons held at one of four constant temperatures: 18, 21, 26, or 30°C. At days chosen based on published extrinsic incubation studies of other flaviviruses, mosquitoes were cold-anesthetized in cohorts of 20 females and then legs and wings were removed and saliva was collected by inserting the proboscis into a capillary tube containing fetal bovine serum for 20 minutes. Individual bodies, legs/wings, and the saliva sample from each mosquito were placed separately in tubes containing cell culture media and homogenized. Viral RNA was extracted and ZIKV viral RNA (vRNA) titers were determined for each body, legs/wings, and saliva sample using RT-qPCR. A cycle threshold (Ct) value ≤ 38 was considered positive for ZIKV RNA. Positive bodies indicated ZIKV infection, positive legs and wings indicated ZIKV dissemination, and positive saliva indicated ZIKV transmission.

Five five-week-old mice were inoculated with 10^5^ Vero plaque forming units (PFU) of ZIKV PR15 via subcutaneous injection, and mosquitoes were allowed to feed on the anesthetized mice two days post-inoculation, at peak viremia [13, 21] (Fig 1). Blood was collected immediately prior to the mosquito feed via each mouse’s submandibular vein (i.e., cheek punch) and centrifuged for five minutes at 6.6 RPM to separate serum from whole blood and stored at -80°C. Blood serum from each mouse was thawed once for Vero cell plaque assay to determine ZIKV viremia. Briefly, cell monolayers were inoculated with 15–30 μL of undilute mouse serum from individual mice mixed with DMEM containing 2% (vol/vol) FBS, and 1% (vol/vol) penicillin/streptomycin. After a one- hour incubation period to allow for viral attachment to cells, an overlay of 0.8% agarose/DMEM was added to cover the cells. Culture plates were incubated at 37°C in 5% CO_2_ for eight days. The cells were then fixed with 4% formaldehyde and stained with 0.05% crystal violet. Plaques were visualized as holes in the Vero cell monolayer and counted to determine PFU values. Two technical replicates were performed for each sample.

### Mosquito infection, dissemination, and transmission

Mice were anesthetized prior to mosquito exposure with a ketamine (VETone Zetamine CIII, 75 mg/kg), xylazine (AnaSed, 10 mg/kg), and acepromazine (AceproJect, 1 mg/kg) solution administered intraperitoneally. Viremic mice were presented at once to >1,200 3–5 day old adult female *Ae*. *aegypti* in a one ft^3^ mesh cage (BugDorm) for 45–60 minutes (Fig 1). Engorged females were randomly sorted via vacuum aspiration into four half-gallon ice cream cartons with 175–190 mosquitoes per carton. Cartons were sorted into four reach-in environmental chambers (Binder KBF 115, Binder, Tuttlingen, Germany); or Darwin IN034, Darwin Chambers, St. Louis, Missouri, USA), each held at a different mean temperature (18°C, 21°C, 26°C, or 30°C), 70–80% relative humidity, and 12:12 hour light:dark cycle. Temperature and humidity in the chambers were measured using HOBO UX100 data loggers (Onset Computer Corporation, Bourne, MA); Binder chambers experienced small fluctuations of up to 2°C around the setpoint mean temperatures, while Darwin chambers held constant temperatures. All mosquitoes had constant access to 10% sucrose after blood-feeding for the remainder of the experiment. Following the infectious bloodmeals, at days chosen based on published extrinsic incubation studies of other flaviviruses [18–20], mosquitoes were cold-anesthetized in cohorts of 20 females at -20°C for five minutes and then legs and wings were removed with forceps while immobilized on ice. Saliva was collected by inserting the proboscis into a capillary tube containing fetal bovine serum (FBS, GenClone, San Diego, CA, USA) for 20 minutes (Fig 1) [22]. Individual bodies, legs/wings, and the saliva sample from each mosquito were stored separately in 2-mL tubes containing 250 μL Dulbecco’s modified eagle medium (DMEM, Gibco) supplemented with 1% penicillin/streptomycin and 10% FBS and either a 5-mm glass bead (bodies, legs/wings) or a 5-mm metal bead (saliva, Qiagen). All samples were stored at -80°C until further processing.

Mosquito tissues and glass capillary tubes containing saliva samples were thawed and homogenized in DMEM by shaking for two to four minutes at 30 shakes/second using a Tissuelyser (Qiagen, Hilden, Germany) and immediately centrifuged at 6.6 RPM for two to three minutes. Viral RNA was extracted using the MagMax Viral RNA Extraction Kit (ThermoFisher, Waltham, MA). A total of 50 μL of homogenate for mosquito tissue and 100 μL of saliva samples were extracted. All RNA extracts were eluted in 50 μL of elution buffer (Buffer EB, Qiagen) and stored at -80°C until further testing.

ZIKV viral RNA (vRNA) titers were determined for each body, legs/wings, and saliva sample using the Taqman Fast Virus 1-Step Master Mix (ThermoFisher) reverse transcription RT-qPCR kit with a previously described ZIKV-specific assay (primers: ZIKV 1086 and ZIKV 1162c, probe: ZIKV 1107-FAM [23]). Two technical replicates were processed for all samples. Samples for which least one technical replicate with a cycle threshold (Ct) value of 38 or below were considered positive for ZIKV vRNA. This limit of detection was determined from prior testing of serially diluted samples of known ZIKV vRNA concentrations with the same extraction and RT-qPCR reagents and protocols and equipment [24]. Ct values were converted to genome copies using standards of known concentration.

The infection rate at each temperature and time point combination was calculated as the number of mosquito bodies positive for ZIKV vRNA by RT-qPCR out of the total number of tested mosquitoes that ingested a bloodmeal. Dissemination and transmission rates were calculated similarly as the number of mosquito leg/wing samples and saliva samples that were vRNA-positive out of the total tested, respectively.

### Statistical analyses

To characterize ZIKV vRNA transmission as a function of time and temperature, a logistic regression model was fitted. The outcome of interest was the proportion of mosquitoes that transmitted ZIKV vRNA, and temperature and days post feeding (dpf) were explanatory variables, with an interaction term included in the model. Temperature and dpf were centered at 26°C and 7dpf prior to model fitting. EIP_50_ was estimated from the fitted curves. To determine whether dissemination titer was predictive of ZIKV vRNA transmission for each temperature, we fitted a logistic regression model for proportion transmitting as a function of dissemination titer (log_10_(genomes)/tissue) with temperature as a categorical covariate. All analyses were done using R version 3.5 [25].

## Results

### Mouse viremias

ZIKV titers in the five mice ranged from 5.3–5.7 log_10_ PFU/mL ZIKV as determined via Vero cell plaque assay (individual mouse titers: 5.3, 5.5, 5.6, 5.6, 5.7 log_10_ PFU/mL). As further verification of the infectious dose, a single blood-fed mosquito collected immediately after feeding imbibed 5.9 log_10_ genomes ZIKV as determined by RT-qPCR.

### Effect of temperature and time on Zika virus infection, dissemination, and transmission

In total, 320 blood-fed mosquitoes survived the duration of the study and were tested for infection, dissemination, and transmission of ZIKV vRNA. In a few samples, a high Ct value >38 or no Ct value was determined in one of two technical replicates where the other technical replicate was ≤38. These samples were considered positive and at the limit of detection by this measurement [19].

**Fig 2:**
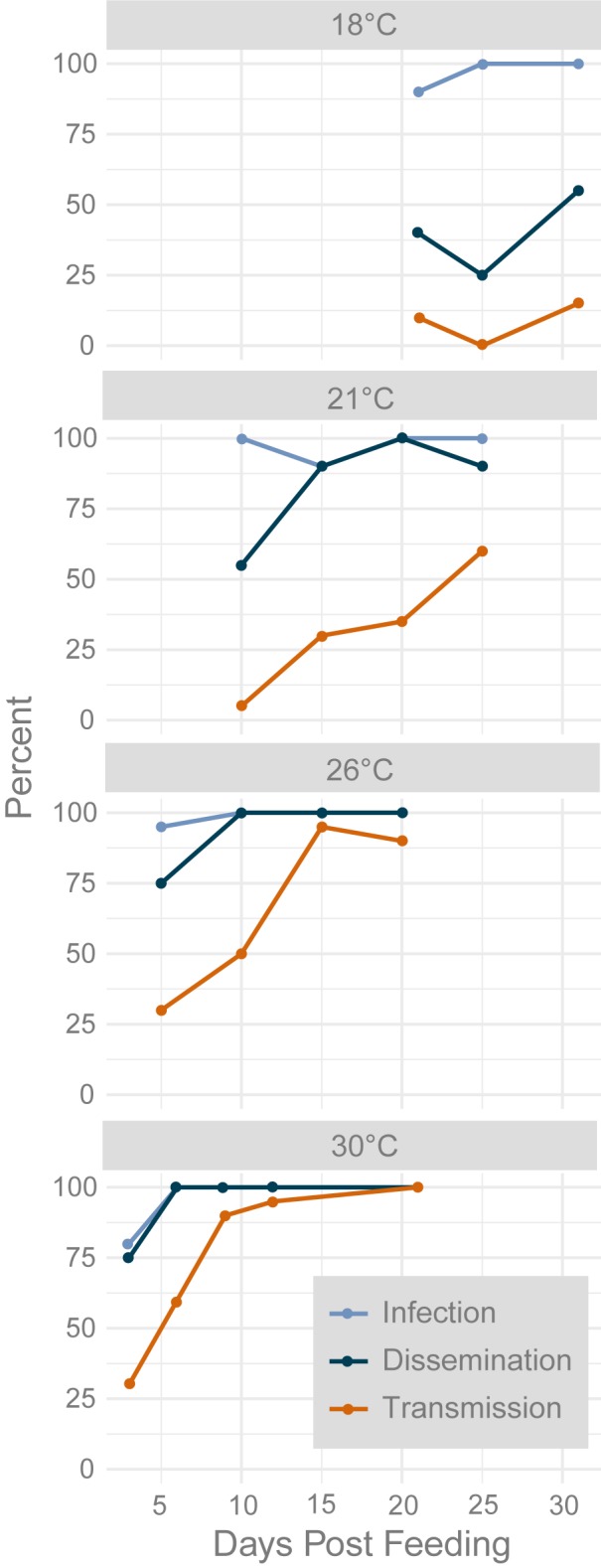
Percent of mosquitoes with infected, disseminated, and transmitted ZIKV vRNA of 20 tested at each temperature and time point combination tested. Samples were processed in duplicate by RT-qPCR. Ct values < 38 in at least one of the duplicates indicated positive vRNA.

Infection, dissemination, and transmission rates increased over time for each temperature, except at 18°C where overall dissemination and transmission was low and inconsistent (Table 1, Fig 2). For all temperatures, the infection rate reached 100% at the first or second time point tested. Dissemination rates reached 100% at 21, 26, and 30°C at the second or third time-point tested. Dissemination rates at 18°C reached a maximum of 55%, which was observed at the last time point of this study (31 dpf). Transmission was detected at the first time point for each temperature: 10% transmitted at 25 dpf at 18°C, 5% transmitted at 10 dpf at 21°C, 30% transmitted at five dpf at 26°C, and 30% transmitted at three dpf at 30°C. Due to low and inconsistent transmission at 18°C, data from the 18°C treatment were omitted from the remainder of the analysis.

**Table 1:** ZIKV vRNA infection, dissemination, and transmission rates by temperature and time point. Samples were processed in duplicate by RT-qPCR. Ct values < 38 in at least one of the duplicates indicated positive vRNA.

Temp (°C)	Days Post Feeding (5.3–5.7 log_10_ PFU/mL)	Infected (%)	Disseminated (%)	Transmitted (%)
18	21	18/20 (90)	8/20 (40)	2/20 (10)
18	25	20/20 (100)	5/20 (25)	0/20 (0)
18	31	20/20 (100)	11/20 (55)	3/20 (15)
21	10	20/20 (100)	11/20 (55)	1/20 (5)
21	15	18/20 (90)	18/20 (90)	6/20 (30)
21	20	20/20(100)	20/20 (100)	7/20 (35)
21	25	20/20 (100)	18/20 (90)	12/20 (60)
26	5	19/20 (95)	15/20 (75)	6/20 (30)
26	10	20/20 (100)	20/20 (100)	10/20 (50)
26	15	20/20 (100)	20/20 (100)	19/20 (95)
26	20	20/20 (100)	20/20 (100)	18/20 (90)
30	3	16/20 (80)	15/20 (75)	6/20 (30)
30	6	20/20 (100)	20/20 (100)	12/20 (60)
30	9	20/20 (100)	20/20 (100)	18/20 (90)
30	12	20/20 (100)	20/20 (100)	19/20 (95)
30	21	20/20 (100)	20/20 (100)	20/20 (100)

### Transmission as a function of time and temperature

The combined effect of time and temperature, represented by the interaction term, was positively associated with the probability of transmission (β_3_ = 0.027, *P* = 0.002). The coefficients from the logistic regression model (Table 2) describe the cumulative probability of transmission over time for any given temperature, according to the formula:

$$ln\left(\frac{p}{1-p}\right)=-0.667+0.299D+0.378T+0.027D\times T$$

where *p* is the probability of transmission, and *D* and *T* are the centered time and temperature covariates, *D = DPF-7* and *T = Temperature-26*, respectively.

**Table 2:** Coefficients from the logistic model for the probability of ZIKV transmission as a function of time and temperature.

Variable	Coefficient (95% CI)	*P-value*
Intercept	-0.667 (-1.16,-0.21)	0.006
DPF	0.299 (0.22,0.39)	<0.001
Temperature	0.378 (0.24,0.53)	<0.001
DPF x Temperature	0.027 (0.01,0.05)	0.002

**Fig 3:**
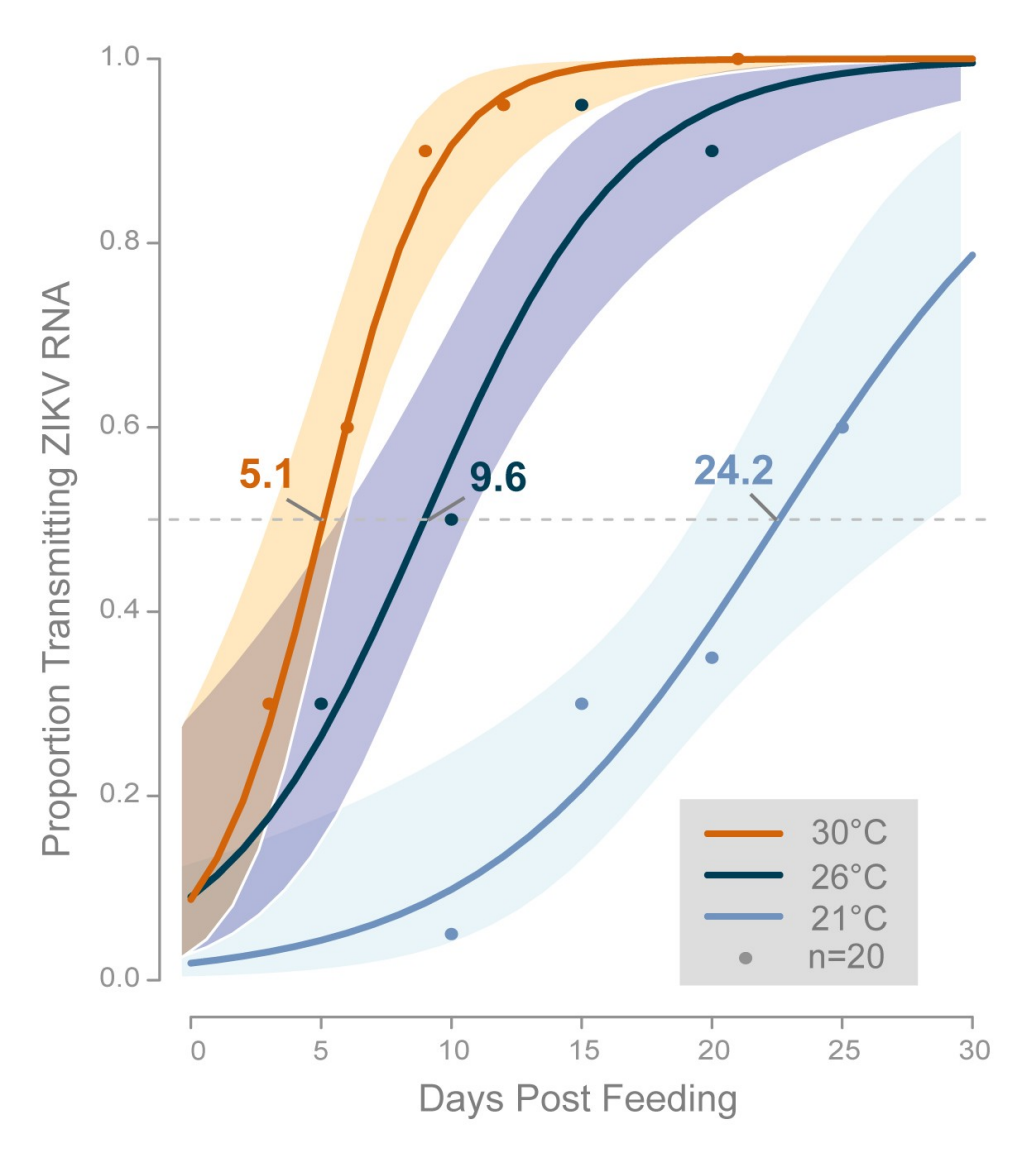
Fitted logistic curves showing the proportions of *Ae*. *aegypti* transmitting ZIKV vRNA over time by temperature. Each point represents the observed proportion of mosquitoes (of 20 tested) that transmitted at each temperature and time-point. The estimated EIP_50_ is indicated for each temperature.

The median time from ZIKV ingestion to transmission of vRNA (median EIP, EIP_50_) was derived from the fitted regression function for each temperature tested and ranged from 5.1 days at 30°C to 24.2 days at 21°C. At 26°C, EIP_50_ was 9.6 days (Fig 3).

### Relationship between disseminated Zika virus titer and probability of transmission

**Fig 4:**
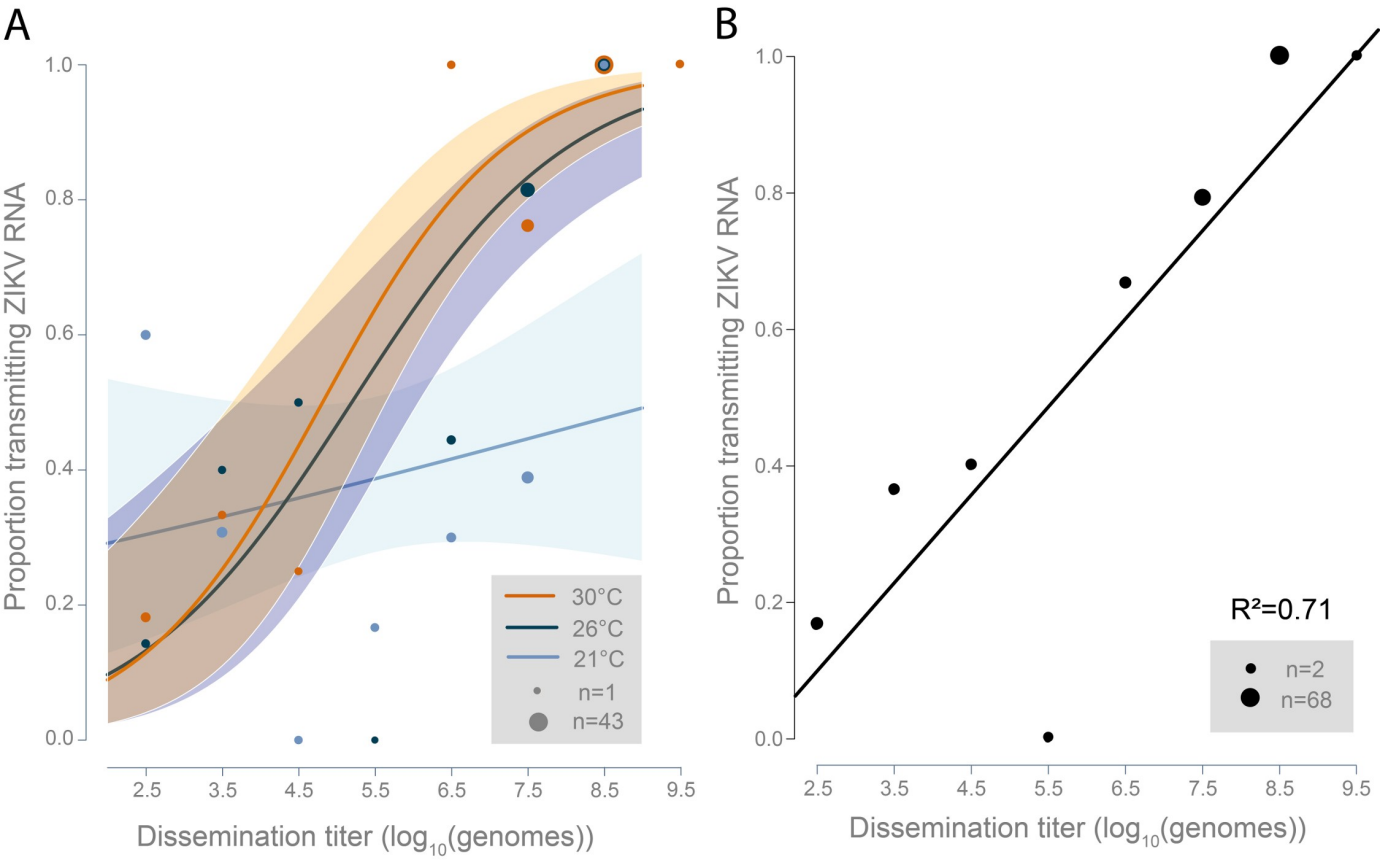
(A) Fitted logistic curves showing the relationship between dissemination titer (log_10_(genomes)) and proportion transmitting ZIKV RNA for each temperature. Dot size indicates the number of mosquitoes within ±.5 log_10_(genomes) of plotted number. (B) Fitted line showing the relationship between dissemination titer (log_10_(genomes)) and proportion transmitting ZIKV RNA for 26 and 30°C combined. Dot size indicates the number of mosquitoes within ±.5 log_10_(genomes) of plotted number.

At 21°C, there was no significant relationship between transmission and dissemination titer (log_10_(genomes)/tissue) (*P* = 0.34, Fig 4A, S1 Table). Dissemination titer was associated with increased probability of transmitting ZIKV vRNA at 26°C and 30°C (Fig 4A, S1 Table). The fitted relationship between transmission and dissemination titer was not significantly different between 26°C and 30°C (*P* = 0.86).

Because there was no significant difference observed between the 26 and 30°C curves, data were pooled for these temperatures, dissemination titer was binned to the nearest .5 log_10_(genomes), and a linear regression model was fitted (Fig 4B). The linear model fit the data well (R^2^ = 0.71). Dissemination titer was a significant predictor of proportion transmitting, such that for each 10-fold increase in dissemination titer, proportion transmitting increases by 13% (*P* = 0.008, S2 Table).

## Discussion

Understanding environmental effects on transmission efficiency of vector-borne pathogens over the seasonal and geographical ranges of their vectors is critical for understanding transmission dynamics and risk. As climate and land use change and invasive mosquitoes are able to establish populations in locations where previously not possible, it is increasingly important to understand this relationship in order to take appropriate measures to protect public health [26–28]. Warmer temperatures are known to shorten the extrinsic incubation of a wide range of mosquito-borne pathogens, but with one recent exception [17], this is not well-characterized for ZIKV outside of the small range from 26–28°C. Other published studies aiming to estimate ZIKV risk have borrowed estimates for other flaviviruses, most notably closely related dengue virus, to mechanistically estimate risk [29,30]. In this study, we determined the effect of a range of constant temperatures on vector competence of *Ae*. *aegypti* from California for a ZIKV strain from Puerto Rico. We demonstrate that increasing temperature shortens EIP and we provide estimates of the combined effects of temperature and time post-feeding on ZIKV transmission potential. These parameters can inform mechanistic models to better understand ZIKV transmission dynamics. Further, we demonstrate that dissemination titer is highly correlated with probability of transmission at warm temperatures, but the relationship is less consistent at the cooler temperatures tested.

In line with results from other mosquito-borne flaviviruses, including dengue virus in *Ae*. *aegypti* and West Nile virus in *Culex tarsalis*, we demonstrate that warmer temperatures shorten the time from an infectious blood meal to transmission within the range tested [17–20,31]. This characterization is especially important where temperatures geographically and seasonally deviate from the 26–28°C range, such as in California and Florida. Infection was high at all temperatures, however dissemination was considerably lower at 18°C, and transmission was rare at 18°C when compared to the other temperatures tested (21°C, 26°C, 30°C). This limitation to midgut escape, dissemination, and overall transmission at 18°C could be due to the effects of low temperature on mosquito immunity [32,33], mosquito physiology [34], and/or viral structure and binding in the mosquito [34,35].

One potential limitation to this study is the use of RT-qPCR to quantify viral RNA. Though it is a common method among vector competence studies, RT-qPCR is more sensitive than other methods such as plaque assay because RT-qPCR quantifies viral RNA, all of which is not necessarily derived from infectious viral particles and may include noninfectious virions. In this study, the use of RT-qPCR across temperatures and timepoints allows for consistent comparison of infection, dissemination, and transmission rates. It is possible that our results underestimate EIP_50_, but we expect any effect would be very slight because two recent studies in our laboratory showed that 71 and 75% of mosquito saliva samples that were positive for ZIKV and WNV, respectively, by RT-qPCR were confirmed positive for infectious virus by plaque assay [13,19].

Origins of the mosquitoes and viral strains used for vector competence and extrinsic incubation studies can affect results [14,16,36]. In this study, we provide estimates for median Zika virus extrinsic incubation periods at the temperatures tested as well as the logistic formula to calculate probability of transmission at any temperature. These estimates and model are solely the result of data from the interactions of one mosquito population and one viral strain assessed in one laboratory. Potential variation in these estimates will become more clearly resolved as more studies are conducted using different mosquito populations and viral strains.

The current standard for detecting transmission by individual mosquitoes is the capillary-tube method, which requires inserting the mosquito proboscis into a small capillary tube with media and waiting >20 minutes for the mosquito to expectorate into the tube [22]. This method is time and resource-consuming and the additional processing required is a key constraint on the potential sample size for vector competence studies. In this study, we determined that dissemination titer is associated with increased probability of transmission of ZIKV vRNA at 26°C and 30°C, but not at 21°C (Fig 4A, S1 Table). These results suggest that dissemination titer quantified by RT-qPCR in controlled laboratory experiments performed under standard, consistent rearing conditions may be a reasonable proxy that is predictive of ZIKV vRNA transmission. Dissemination titer from legs and wings was considered instead of body titer because this ensures that the virus has overcome the midgut escape barrier and because body titer was high at the first time point tested for each temperature and plateaued quickly compared to dissemination titer (S1 Fig). The lack of correlation at 21°C could be a result of the time it takes the virus to reach the salivary glands via hemolymph-mediated viral circulation at this cooler temperature being faster than binding and replication in the hemolymph or other secondary organs, however this was not been tested in the current study. Based on the data, these findings do not extend to field applications but could guide optimization of a laboratory method for assessing vector competence around the 26–30°C range tested without the need for relying on the capillary tube method.

The results of this study encompassed the inherent biological variation in the large number of bloodfed *Ae*. *aegypti* females that were sourced from a low-generation (F4) colony and fed on five viremic mice. Temperature treatments were not replicated because temperature is an abiotic variable that was verified by both the built-in chamber thermometer and secondary data loggers placed inside the chambers. Due to differences in the chambers available for our study, two of our chambers varied slightly (up to 2°C) around the mean temperatures of 18 and 21°C, whereas the other two chambers maintained constant temperatures of 26 and 30°C. We do not believe this caused any bias in our findings because a previous study with another flavivirus in our lab showed that small to moderate diurnal temperature ranges–larger than those in this study–did not alter extrinsic incubation periods compared to constant-temperature treatments [37]. Also, an earlier study on infection and dissemination of dengue virus in *Ae*. *aegypti* found that even large daily temperature fluctuations of 20°C affected only the proportion of mosquitoes with midgut infections but did not alter dissemination compared to constant temperatures [38]. In our study, infection rates were consistently high across all temperature treatments, giving further confidence that chamber assignments did not alter our findings.

**Fig 5:**
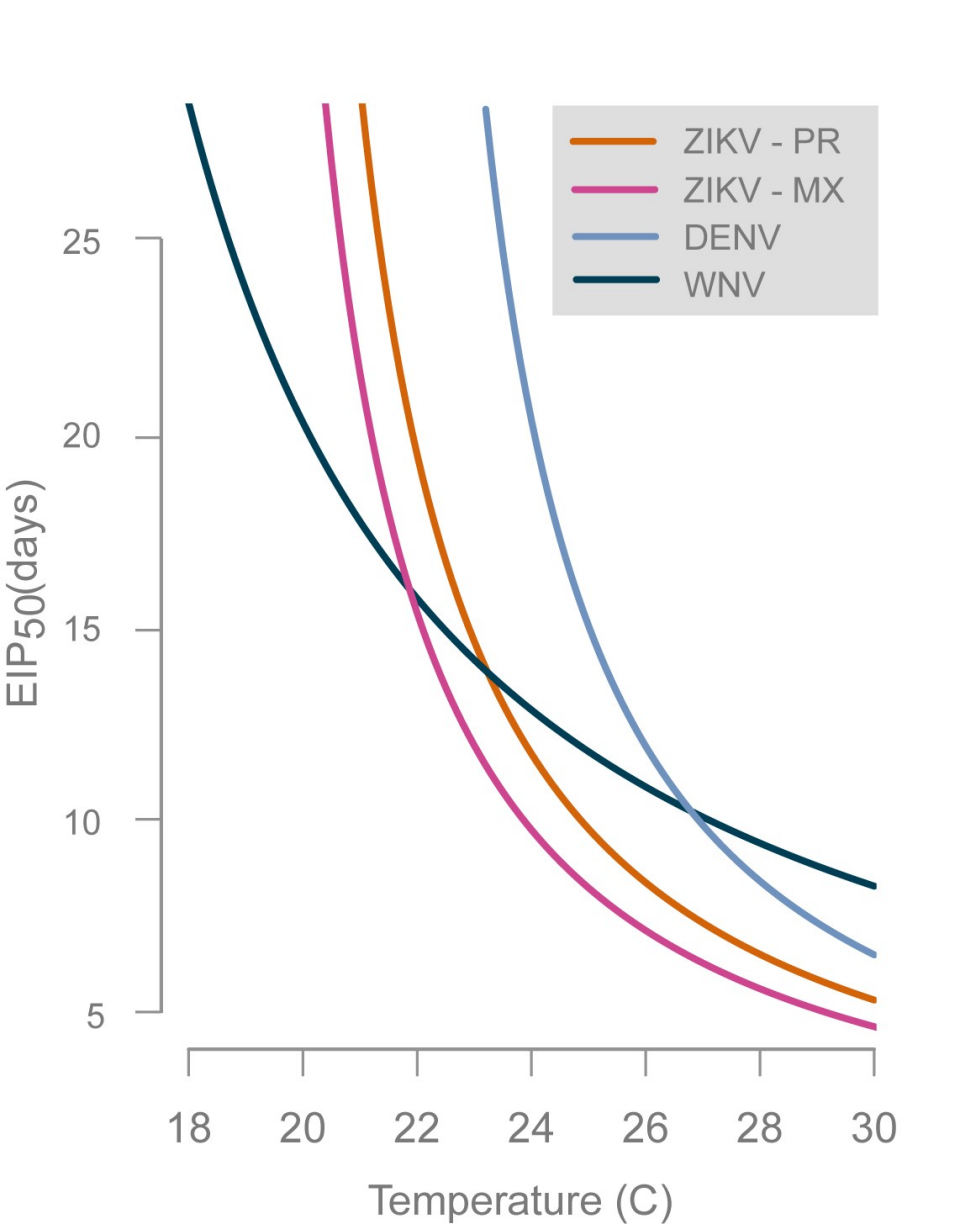
Fitted curves showing the median EIP of the flaviviruses ZIKV, dengue virus (DENV), and West Nile virus (WNV) over a range of temperatures. ZIKV–PR is the Puerto Rico strain used in this study, whereas ZIKV–MX is a Mexican strain used by Tesla et al. 2018.

The relationship between temperature and ZIKV EIP in *Ae*. *aegypti* reported in this study was compared to published EIPs of other mosquito-borne viruses using linear regression (Fig 5). West Nile virus data in *Cx*. *tarsalis* from two previous studies in our laboratory using the same methods were used [18,19]. Data on dengue virus from a meta-analysis of EIP studies in *Ae*. *aegypti* was used [20]. Finally, we compared estimates for ZIKV EIP from the current study using a 2015 ZIKV outbreak strain from Puerto Rico (ZIKV–PR) to recently published data using a 2016 ZIKV outbreak strain from Tapachula, Chiapas, Mexico (ZIKV–MX) and a sympatric *Ae*. *aegypti* population [17]. EIP_50_ was calculated at timepoints spanning 18 to 30°C using the fitted models reported in the respective studies, including the current study. We fitted linear models to extrinsic incubation rates (1/EIP) for each virus (Fig 5).

The regression curves (Fig 5) suggest that WNV in *Culex tarsalis* has a broader range of temperatures at which transmission is feasible when compared to ZIKV and DENV, however at higher temperatures ZIKV and DENV transmission are faster than WNV. Both ZIKV EIP curves have a similar shape to DENV, however the curves suggest ZIKV EIP is shorter across temperatures when compared to DENV in *Ae aegypti*. Finally, the EIP curve determined in this study was remarkably similar in shape to another published curve for ZIKV, although our study’s median EIP was 0.7–1.3 d longer for temperatures from 30 to 26°C where transmission was most efficient and approximately 4.0 d longer at 22°C. It is not possible to disentangle the possible explanations for the differences because our study differed from [17] in viral strain, mosquito strain, method of mosquito infection (viremic mouse vs. artificial membrane feeder), method of ZIKV detection (RT-qPCR vs. plaque assay), and calculation of EIP (transmission out of total vs. transmission out of infected). Overall, it is interesting to note that both ZIKV studies (ours and [17]) have shorter EIPs across temperatures when compared to DENV, which may partially explain the rapid ZIKV spread throughout the Americas. It is important to note that both ZIKV EIP studies have used outbreak strains from the 2015–2016 Latin American Zika epidemic. We do not conclude that ZIKV EIP will always be shorter than DENV and it is possible that non-outbreak Zika virus strains or populations of *Ae*. *aegypti* from outside of the Americas will yield different results. The effect of temperature on ZIKV EIP will be better resolved as more data is collected on using different strains of ZIKV and populations of *Ae*. *aegypti*.

In summary, this study determined the effects of temperature and time on infection, dissemination, transmission, and EIP of a Puerto Rican strain of ZIKV in *Ae*. *aegypti* from central California. This combination has relevance to the potential for ZIKV to be transmitted within the continental U.S. We fitted a logistic regression model to determine ZIKV vRNA transmission as a function of time and temperature and estimated EIP_50_ from the fitted curves; we report that as temperature increases, EIP decreases within the temperature bounds tested. Further, we demonstrate that dissemination titer is correlated with probability of transmission at high temperatures at standard rearing conditions, but not at 21°C. This method could be optimized for use a laboratory method to estimate relative transmission rates without using the capillary tube method. Finally, our data, in addition to data from a similar study, suggest that ZIKV EIP may be accelerated compared to dengue virus over the range of temperatures tested. This information is critical for modeling ZIKV transmission dynamics to more accurately understand geographic and seasonal limits of ZIKV transmission risk.

## Supporting information

S1 Fig ZIKV infection titer (top panel) and dissemination titer (bottom panel) over time for each temperature.
(TIFF)

S1 Table Coefficients from the logistic regression model for the probability of ZIKV transmission as a function of dissemination titer and temperature. 21°C was the referent group. Coefficients are on the log odds (logit) scale.
(DOCX)

S2 Table Coefficients from the linear model for the probability of ZIKV transmission as a function of dissemination titer. Due to no significant difference in the logistic curves for 26 and 30°C, data were pooled and dissemination titer was binned to the nearest 0.5 log_10_(genomes).
(DOCX)
