# Supplementary figures and images for: Impact of temperature on the extrinsic incubation period of Zika virus in Aedes aegypti
Source: PLoS Negl Trop Dis. 2020 Mar 18;14(3):e0008047. doi: 10.1371/journal.pntd.0008047 (PMC7105136; doi:10.1371/journal.pntd.0008047)

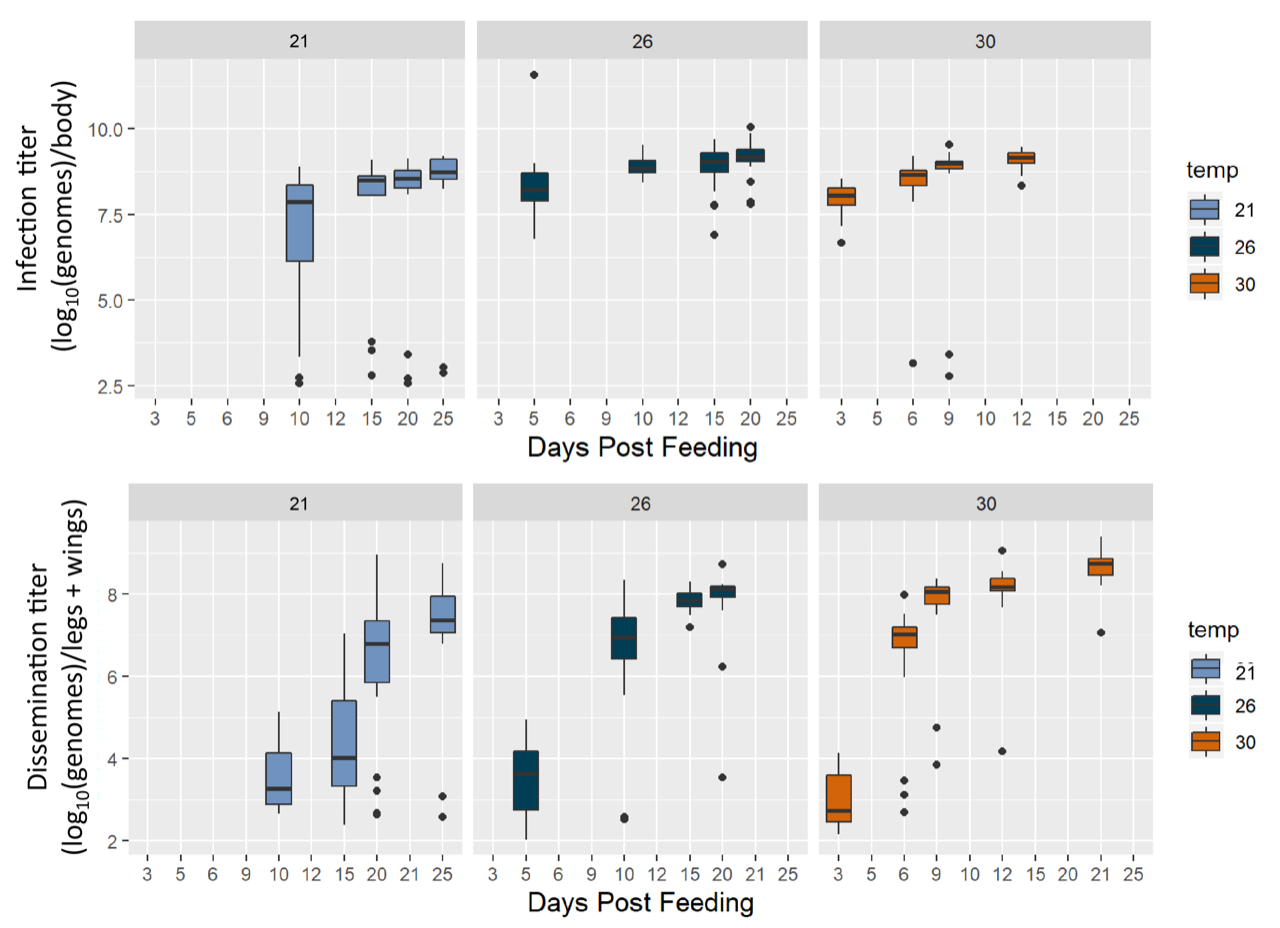

Supplement: S1 Fig — ZIKV infection titer (top panel) and dissemination titer (bottom panel) over time for each temperature. (TIFF) [file pntd.0008047.s001.tiff]
