# Supplementary material for: Impact of temperature on the extrinsic incubation period of Zika virus in Aedes aegypti
Source: PLoS Negl Trop Dis. 2020 Mar 18;14(3):e0008047. doi: 10.1371/journal.pntd.0008047 (PMC7105136; doi:10.1371/journal.pntd.0008047)
Supplement: S1 Table — 21°C was the referent group. Coefficients are on the log odds (logit) scale. (DOCX) [file pntd.0008047.s002.docx]

| **Variable** | **Coefficient (95% CI)** | ***P*-value** |
| --- | --- | --- |
| Intercept | -1.13 (-2.62, 0.36) | 0.136 |
| Dissemination Titer | 0.12 (-0.13, 0.37) | 0.340 |
| Temperature (26°C) | -2.50 (-5.10, 0.10) | 0.059 |
| Temperature (30°C) | -2.84 (-5.28, -0.40) | 0.022 |
| Dissemination Titer x Temperature (26°C) | 0.58 (0.17, 0.98) | 0.006 |
| Dissemination Titer x Temperature (30°C) | 0.70 (0.31, 1.10) | <0.001 |

S1 Table: Coefficients from the logistic regression model for the probability of ZIKV transmission as a function of dissemination titer and temperature. 21°C was the referent group. Coefficients are on the log odds (logit) scale.
