# Supplementary material for: Impact of temperature on the extrinsic incubation period of Zika virus in Aedes aegypti
Source: PLoS Negl Trop Dis. 2020 Mar 18;14(3):e0008047. doi: 10.1371/journal.pntd.0008047 (PMC7105136; doi:10.1371/journal.pntd.0008047)
Supplement: S2 Table — Due to no significant difference in the logistic curves for 26 and 30°C, data were pooled and dissemination titer was binned to the nearest 0.5 log10(genomes). (DOCX) [file pntd.0008047.s003.docx]

| Variable | Coefficient (95% CI) | *P-value* |
| --- | --- | --- |
| Intercept | -0.23 (-0.75,0.30) | 0.332 |
| Dissemination titer | 0.13 (0.05, 0.21) | 0.008 |

**S2 Table:** Coefficients from the linear model for the probability of ZIKV transmission as a function of dissemination titer. Due to no significant difference in the logistic curves for 26 and 30°C, data were pooled and dissemination titer was binned to the nearest 0.5 log(genomes).
